# Xylitol, Mitochondrial Plasticity, the Warburg Effect, and Oral Pathobiont-Associated Immune Evasion in Cancer Hypothesis

**DOI:** 10.3390/ijms27146130

**Published:** 2026-07-09

**Authors:** Mark Cannon, John Peldyak

**Affiliations:** 1 Ann & Robert H. Lurie Children’s Hospital of Chicago, Northwestern University Feinberg School of Medicine, Chicago, IL 60611, USA; 2 Independent Researcher, Mount Pleasant, MI 48858, USA

**Keywords:** xylitol, Warburg effect, cancer metabolism, lactate, mitochondria, cancer stem cells, oral microbiome, gut microbiome, Streptococcus mutans, pentose phosphate pathway, metabolic disease, xylitol dehydrogenase, cristae, anti-adhesion, senescent immune cells, mucositis

## Abstract

The Warburg effect is better understood as regulated metabolic plasticity rather than mitochondrial failure. Many malignant cells retain functional mitochondria while increasing aerobic glycolysis, lactate production, and redox remodeling to support growth, immune escape, and adaptation to microenvironmental stress. Within the context of the cancer microenvironment, this review examines xylitol as a hypothetical metabolic modifier within a broader host-microbe-mitochondria framework. Xylitol, a five-carbon sugar alcohol, is derived endogenously through the pentose phosphate pathway (PPP) and the glucuronate–xylulose pathway, and is metabolized efficiently in humans, rats, and pigs through xylitol dehydrogenase (XDH) in hepatic mitochondria and the cytosol; whereas, it is less tolerated by obligate carnivores who lack this enzyme. Preclinical studies show that partial substitution of glucose with xylitol can reduce proliferation and glycolytic markers in oral squamous carcinoma models, and preliminary studies link xylitol to glutathione depletion, endoplasmic reticulum (ER) stress, autophagy-associated death, and altered tumor metabolomics. On the other hand, oral pathogens such as *Fusobacterium nucleatum* and *Porphyromonas gingivalis* promote tumor stemness, extracellular vesicle signaling, metastasis, and immune evasion. In addition, *Streptococcus mutans*, the primary cariogenic pathogen, contributes to systemic bacteremia and epithelial–mesenchymal transition. Oral and gut microbiomes modulate macrophage polarization, T cell activity, and the senescence-associated secretory phenotype (SASP), possibly promoting cancer immune evasion. The anti-adhesive properties of xylitol may limit pathogen attachment to immune cell receptors, reducing the generation of pro-tumorigenic senescent immune cells. Xylitol also offers metabolic benefits, a low glycemic index, partial insulin-independent metabolism, and potential diabetes-prevention activity that are relevant, considering the established link between metabolic disease and cancer risk. A recent study reported that higher levels of endogenous xylitol were associated with adverse cardiovascular events, but confirmation of this requires large scale prospective studies. The evolutionary dietary context of MIS 6, during which hominin populations in sub-Saharan Africa depended on polyol-rich underground storage organs, provides a biological basis for human tolerance of xylitol. As a result, we hypothesize that xylitol may be a context-dependent metabolic modifier within an integrated host–microbe–mitochondria–cancer stem cell network.

## 1. Introduction

Cancer metabolism involves dynamic allocation of carbon, reducing equivalents, and biosynthetic precursors rather than a binary choice between glycolysis and oxidative phosphorylation (OXPHOS). Malignant cells often maintain mitochondrial function while increasing aerobic glycolysis to support biomass production, lactate signaling, redox adaptation, and microenvironmental conditioning [1,2]. Cancer stem cells (CSCs) can shift between glycolytic and oxidative states and exploit mitochondrial biogenesis, fission–fusion balance, mitophagy, and redox buffering to survive therapeutic and nutritional stress [3].

Xylitol is a five-carbon sugar alcohol with established dental applications [4,5] and emerging preclinical interest in cancer metabolism. Partial substitution of glucose with xylitol reduces proliferation in models of oral squamous carcinoma, lung cancer, and melanoma, with associated changes in glycolytic flux, ATP production, glutathione homeostasis, ER stress, and autophagy [4,5,6,7,8,9]. Xylitol arises endogenously in the liver through the glucuronate–xylulose pathway and the PPP [10,11,12,13,14], and XDH, also termed L-iditol dehydrogenase or sorbitol dehydrogenase, catalyzes the interconversion of xylitol and D-xylulose in both mitochondrial and cytosolic compartments [15,16,17].

The oral pathobionts *F. nucleatum*, *P. gingivalis*, and *S. mutans*, together with the gut microbiome, have been linked to cancer progression, immune evasion, and systemic metabolic disease [18,19,20,21,22,23,24,25]. Mitochondrial transfer, lactate signaling, cristae architecture, and the immunological consequences of senescent immune cells provide converging rationale for evaluating xylitol in an integrated host–microbe–mitochondria–CSC network [26,27,28,29].

Human populations who survived the sub-Saharan African megadrought of Marine Isotope Stage 6 (MIS 6, ~190–130 ka BP) depended on polyol-rich underground storage organs (USOs) [30,31], selecting for efficient XDH-based xylitol metabolism—present in omnivores, including humans, rats, and pigs but absent in carnivores such as dogs [32,33].

## 2. Scope

This narrative review covers six intersecting topics: xylitol biology, metabolism, and evolutionary context; cancer metabolic plasticity, including the Warburg effect and lactate signaling; the hepatic PPP and glucuronate–xylulose pathways as endogenous xylitol sources; xylitol interactions with mitochondrial cristae and OXPHOS; oral and gut pathobionts, immune evasion, and senescent immune cells; and the metabolic disease–cancer link [8,9,34,35,36,37]. Published xylitol studies were specifically included. Translational statements are framed as testable hypotheses rather than clinical recommendations. The literature search was conducted in PubMed/MEDLINE and Scopus, querying records from 1956 through April 2026 using the terms ‘xylitol,’ ‘Warburg effect,’ ‘cancer metabolism,’ ‘oral microbiome,’ ‘*Fusobacterium nucleatum*,’ ‘*Porphyromonas gingivalis*,’ ‘mitochondrial plasticity,’ and ‘senescence-associated secretory phenotype’; studies directly evaluating xylitol or closely related polyols were prioritized, supplemented by mechanistic reviews from cancer biology, microbiology, and immunology. The central hypothesis of this review is that xylitol, through converging metabolic, antimicrobial, and anti-adhesive mechanisms, may act as a context-dependent modifier of tumor-supporting processes, including aerobic glycolysis, oral pathobiont-mediated immune evasion, and SASP-driven tumor microenvironment conditioning, warranting systematic evaluation in defined tumor–microbe–immune contexts; current evidence remains largely preclinical and mechanistically theoretical.

## 3. Warburg Metabolism, Lactate Signaling, and Mitochondrial Competence

Warburg observed in 1956 that cancer cells preferentially use aerobic glycolysis even in the presence of adequate oxygen [38]. Many tumors nevertheless retain functional mitochondria and operate glycolysis and OXPHOS in parallel, adapting to oxygen gradients, substrate availability, and immune pressure [1,2]. Lactate is not merely a glycolytic waste product; it functions as a carbon shuttle and immunomodulatory signal that impairs antigen presentation, T cell and NK-cell activity, and myeloid polarization [27,39]. Lysine lactylation links glycolytic flux to gene regulation, immune suppression, stemness, and therapy resistance [40,41]. A glycolytic perturbation—such as partial glucose-to-xylitol substitution—may not suppress tumor growth if cells compensate through OXPHOS, fatty acid oxidation, or mitochondrial acquisition from neighboring cells [1,2,26]. Conversely, glycolysis-dependent tumors with limited mitochondrial reserve may be selectively vulnerable. Cristae morphology, regulated by OPA1 and the MICOS complex, shapes electron-transport-chain (ETC) supercomplex assembly and respiratory output [42], and OPA1-dependent cristae organization has been identified as a selective vulnerability in metastatic breast cancer cells [43,44]. Importantly, tumor metabolic phenotypes vary substantially: OXPHOS-dominant malignancies, including certain B cell lymphomas, renal cell carcinomas, pancreatic ductal adenocarcinomas, and uveal melanomas, would be predicted to show minimal responses to glycolytic perturbation by xylitol [1,2]. Any potential metabolic effects of xylitol must therefore be interpreted in the context of tumor-specific metabolic dependence, and future studies should stratify models by reliance on glycolysis versus OXPHOS.

## 4. Xylitol Metabolism: Endogenous Pathways and Species Differences

### 4.1. Hepatic Glucuronate–Xylulose and Pentose Phosphate Pathways

**Figure 1:**
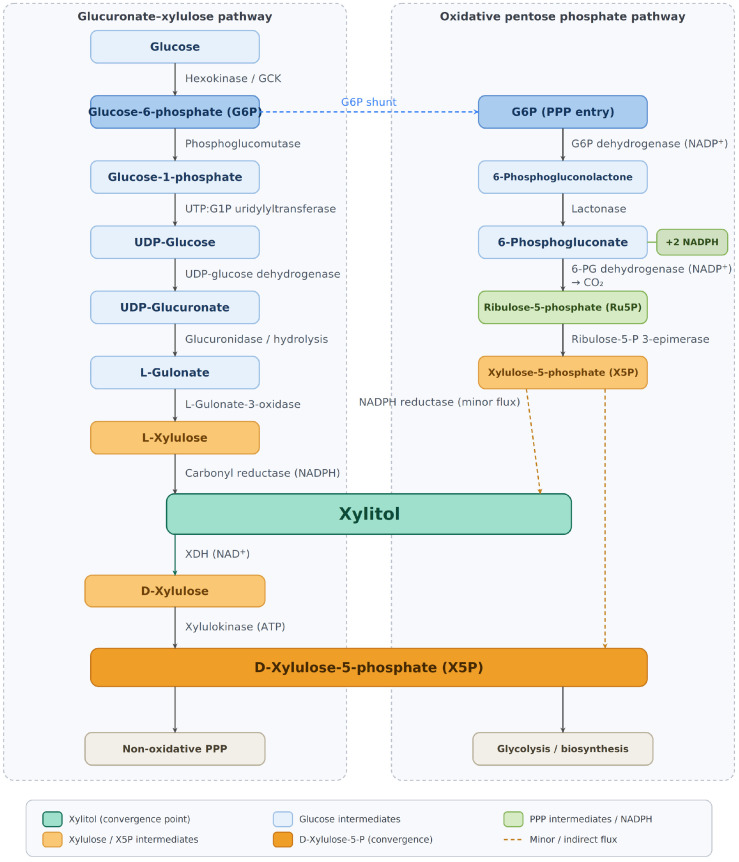
Hepatic glucuronate–xylulose and pentose phosphate pathways converging on xylitol. G6P enters the oxidative PPP to produce ribulose-5-phosphate and NADPH. In parallel, G6P flows through UDP-glucuronate to L-xylulose, which is reduced to xylitol by NADPH-dependent carbonyl reductase. XDH oxidizes xylitol to D-xylulose; xylulokinase phosphorylates D-xylulose to X5P, which enters the non-oxidative PPP.

Xylitol is produced endogenously in the liver (see Figure 1) via the glucuronate–xylulose pathway: glucose-6-phosphate → glucose-1-phosphate → UDP-glucose → UDP-glucuronate → L-gulonate → L-xylulose → xylitol (NADPH-dependent reduction) → D-xylulose (via XDH) → D-xylulose-5-phosphate → PPP [12,14]. The oxidative PPP generates ribulose-5-phosphate and NADPH from glucose-6-phosphate [13]. D-xylulose-5-phosphate serves as the convergence point, entering the non-oxidative PPP and feeding glycolytic intermediates [13]. Under hyperglycemic conditions, flux through the glucuronate–xylulose pathway is increased, potentially increasing endogenous xylitol production [45].

### 4.2. Xylitol Dehydrogenase in Human Mitochondria

XDH, classified within the NAD+-dependent alditol oxidoreductase and aldo-keto reductase superfamilies, catalyzes xylitol oxidation to D-xylulose [15,16,17]. Jeffery and Jörnvall demonstrated sorbitol dehydrogenase/XDH activity in the human liver, consistent with overlapping substrate specificities of this superfamily [17]. Wermuth and von Wartburg characterized a broad-specificity carbonyl reductase in the human brain that accepts xylitol-related substrates [15]. Within mitochondria, XDH-mediated NAD+ consumption and NADH production can alter the mitochondrial redox poise (NADH/NAD+ ratio), potentially modulating ETC activity, ROS output, and cristae-dependent supercomplex stability [16,42]. This indirect link between xylitol metabolism and cristae-dependent OXPHOS warrants direct experimental investigation. Direct experimental evidence linking xylitol exposure to ultrastructural mitochondrial changes in cancer cells is currently lacking; the proposed mechanism remains a testable hypothesis. Approaches that could evaluate this link include: (1) Seahorse extracellular flux analysis (OCR/ECAR ratios) under xylitol versus equimolar glucose/osmolarity controls; (2) transmission electron microscopy for cristae ultrastructure scoring; (3) blue native PAGE for ETC supercomplex profiling; (4) stable-isotope tracer studies using U-13C5-xylitol to map carbon flux through XDH and the non-oxidative PPP; and (5) mitochondrial membrane potential imaging with concurrent ROS quantification in cancer cell lines stratified by XDH expression.

### 4.3. Species-Dependent Metabolism

Humans, rats, and selected pig breeds express hepatic XDH activity (see Table 1) and efficiently clear xylitol by entering D-xylulose into the PPP or via gluconeogenesis [32]. Xylitol research into dietary supplementation with xylitol in mice demonstrates a gut microbiome shift but no change in lipid metabolism [46]. In contrast, domestic dogs express little hepatic XDH; ingested xylitol stimulates a potent insulin secretory response, producing life-threatening hypoglycemia and hepatotoxicity at doses above 0.1 g/kg [33].

## 5. Evolutionary Context: MIS 6 and Polyol-Rich Diets

This section provides background information on species-specific XDH expression and the basis of human polyol tolerance. The evolutionary argument is acknowledged to be inferential and does not constitute evidence for population-level cancer protection from xylitol.] MIS 6 (~190,000–130,000 years ago) was among the most severe glacial episodes of the Pleistocene, marked by sub-Saharan African megadrought and contraction of surface water and C3 plant foods [31]. Early *Homo sapiens* populations concentrated in coastal refugia and inland areas where USOs of geophytic plants became critical fallback foods [30]. Laden and Wrangham proposed that USOs—corms, tubers, and rhizomes of species including *Hypoxis hemerocallidea* (the African potato), *Dioscorea* spp., and *Cyperus* spp.—provided starch, fiber, and polyols including D-xylose and xylitol [30]. Potts’s variability-selection hypothesis holds that climatic instability selected for physiological flexibility rather than dietary specialization [31], implying that recurring exposure to dietary polyols selected for efficient XDH-based metabolism. This context suggests that moderate xylitol intake—well within the 40–70 g/day range shown to be safe in clinical trials [47]—represents a physiologically familiar substrate rather than a xenobiotic one.

## 6. Oral Microbiome, *Streptococcus mutans*, and Cancer

### 6.1. Streptococcus Mutans: Caries Pathogen to Systemic Risk

*Streptococcus mutans* is the principal cariogenic pathogen, defined by its ability to ferment sugars to lactic acid, synthesize glucans via glucosyltransferases, and adhere to hard and soft oral tissues through SpaP/P1, glucan-binding proteins, and collagen-binding protein Cnm. Loesche further established *S. mutans* as a keystone caries pathogen [48]. Cnm-expressing strains invade vascular endothelial cells via collagen binding, a mechanism associated with the risk of infective endocarditis [49]. Meurman and colleagues documented associations between oral streptococcal burden and cardiovascular disease [50]. Tsai et al. demonstrated that *S. mutans* promotes epithelial–mesenchymal transition (EMT) in oral squamous cell carcinoma cells through direct bacterial-host contact and secreted factors, facilitating local invasion [51], a hallmark shared with *F. nucleatum* and *P. gingivalis* [18,21]. Bowen and colleagues showed that the glucan–fructan matrix produced by *S. mutans* scaffolds polymicrobial biofilms, amplifying the inflammatory and invasive potential of co-resident periodontal pathogens [52].

### 6.2. Xylitol Inhibition of Oral Pathogens

Xylitol enters *S. mutans* via the phosphoenolpyruvate-phosphotransferase system, where it is phosphorylated to xylitol-5-phosphate—a non-metabolizable intermediate that accumulates and is futile-cycled, depleting cellular energy and selecting for less adhesive strains [53]. This inhibits acid production, glucosyltransferase activity, glucan synthesis, and biofilm formation [34,47,53]. Mäkinen et al. reported 30–60% reductions in caries in clinical trials of xylitol-containing gums [47]. Söderling showed that maternal xylitol use reduces mother-to-child transmission of *S. mutans*, reshaping oral ecology toward commensal-dominant profiles [53]. Xylitol also inhibits adhesion of non-typeable *Haemophilus influenzae* and *Streptococcus pneumoniae* to nasopharyngeal epithelium via competitive displacement of D-mannose/D-galactose receptor ligands [54]. These findings are consistent with the goals of the phase III NCT07022678 trial evaluating xylitol dental wipes in pediatric AML [55]. Han et al. provided direct evidence that xylitol suppresses *P. gingivalis* LPS-induced IL-1β, IL-6, and TNF-α expression in macrophage models [56]. Cannon and Stevenson documented the effects of xylitol on *S. mutans*, *P. gingivalis*, *F. nucleatum*, and *Candida* spp. in a recent article [35].

## 7. Gut and Oral Microbiome in Cancer and Immune Evasion

### 7.1. The Microbiome–Cancer Nexus

Sepich-Poore et al. demonstrated that tumor-resident bacteria are detectable across diverse cancer types and provide diagnostic microbiome signatures [23]. Garrett reviewed how *F. nucleatum*, enterotoxigenic *Bacteroides fragilis*, and other gut bacteria promote colorectal cancer through metabolite production, immune subversion, and direct epithelial invasion [24]. Schmidt et al. identified enrichment of *Fusobacterium*, *Prevotella*, and *Peptostreptococcus* in oral cancer tumor niches [25]; oral bacteria can translocate to the colon, pancreas, liver, and lung through bacteremia and aspiration [23,25].

### 7.2. Immune Evasion and Cellular Senescence

*F. nucleatum’s* Fap2 protein binds TIGIT on NK and T cells, preventing cytotoxic killing of tumor cells [20]. Wang et al. showed that *F. nucleatum* colonizes colonic crypts and induces tumor stem-cell neogenesis through Wnt/β-catenin activation [18]. *P. gingivalis* protects oral cancer cells from macrophage killing by polarizing macrophages toward M2 states [21]. Lin et al. described a macrophage communication network linking *P. gingivalis* infection to systemic inflammatory disease [22], and Muñoz-Medel et al. implicated *P. gingivalis* in immune evasion in gastric cancer [57]. Campisi and d’Adda di Fagagna established that senescent cells generate the SASP—IL-6, IL-8, MMP-3, VEGF—which promotes tumor progression and immunosuppression [28]. Coppé et al. demonstrated that SASP drives tumor progression and immune evasion in adjacent cells [29]. Chronic sub-threshold activation of pattern recognition receptors on macrophages and neutrophils by oral pathobionts can drive stress-induced immune cell senescence, depleting surveillance capacity and enriching for immunosuppressive SASP-secreting populations. It is important to note that the link between oral pathobiont-driven PAMP stimulation and immune cell senescence in the cancer context remains a proposed mechanism; direct experimental evidence is currently lacking and is identified as a priority for future investigation.

### 7.3. Anti-Adhesive Properties of Xylitol and Senescent Immune Cell Prevention

Oral pathobionts attach to macrophages, dendritic cells, and neutrophils via lectin–carbohydrate interactions, binding bacterial fimbriae to D-mannose and D-galactose receptors [54]. As a structural analog of these carbohydrate ligands, xylitol can competitively occupy these receptors, reducing pathogen adhesion without triggering full pro-inflammatory signaling [53,54]. By reducing chronic, repetitive PAMP stimulation of immune cells, xylitol may preserve immune cell functional longevity and limit SASP-driven pro-tumorigenic signaling—within a mechanistic framework [37]. This is consistent with xylitol’s documented suppression of *P. gingivalis*-induced cytokine production [56] and with published data on inhibition of oral pathobionts [35,36].

## 8. Metabolic Benefits of Xylitol and the Metabolic Disease–Cancer Link

Xylitol has a glycemic index of ~7–13, compared with 65 for sucrose and 100 for glucose, resulting in minimal postprandial spikes in glucose and insulin [58,59]. Hepatic xylitol uptake via GLUT2 is converted by XDH to D-xylulose and enters the PPP without insulin-dependent GLUT4 transport or hexokinase-mediated phosphorylation in peripheral tissues [13,45]. Islam documented associations between xylitol supplementation (20–40 g/day) and reductions in HbA1c and postprandial glucose, as well as improved lipid profiles, in animal and human studies [60].

It is noted that reduced cariogenic biofilm has downstream systemic anti-inflammatory effects that may benefit metabolic health [58,61,62]. Obesity, type 2 diabetes, and metabolic syndrome (see Table 2) are established risk factors for colorectal, endometrial, breast, liver, pancreatic, and kidney cancers [62,63]. Cowey and Hardy described how chronic hyperinsulinemia and elevated IGF-1 promote cancer cell proliferation via PI3K/AKT/mTOR, while adipokines and systemic inflammation create a tumor-permissive microenvironment [62]. Gallagher and LeRoith demonstrated that hyperinsulinemia reduces IGF-binding protein production, increasing bioavailable IGF-1 to tumor cells [63]. High ambient glucose further exacerbates the Warburg phenotype and reinforces lactate-mediated immunosuppression [1,2,40,41]. By reducing glycemic load, xylitol may interrupt this reinforcement loop.

### Xylitol and Cardiovascular Risk: Contextual Considerations

A balanced evaluation of xylitol’s metabolic profile requires acknowledgment of reported cardiovascular safety signals. Witkowski et al. [64] reported that elevated circulating xylitol concentrations were associated with increased platelet aggregation and cardiovascular event risk in a large observational cohort, and that exogenous xylitol supplementation enhanced platelet reactivity in ex vivo and murine thrombosis models. These findings warrant attention and have been the subject of significant scientific discussion [65]. Several contextual factors are relevant to their interpretation in the current review: (1) the circulating xylitol measured in the Hazen study reflects endogenous production via the glucuronate–xylulose pathway under conditions of metabolic stress or hyperglycemia, rather than exogenous dietary intake (the subjects were fasting); (2) the doses used in platelet-reactivity experiments were pharmacological rather than dietary; (3) the observational associations are subject to confounding by shared metabolic disease risk factors; and (4) no prospective randomized trial has demonstrated a causal cardiovascular harm from dietary xylitol at established clinical doses (20–70 g/day). Nonetheless, until this question is resolved by prospective evidence, the potential prothrombotic signal associated with high-dose xylitol supplementation should be explicitly communicated, and caution may be warranted in patients with established cardiovascular disease.

**Table 2:** Xylitol: metabolic and chronic disease-relevant effects.

Disease Area	Mechanism	Key Findings	References
Dental caries	Inhibits *S. mutans* glucan synthesis; futile cycling; selects non-adhesive strains	30–60% caries reduction in clinical trials; plaque weight reduction	[34,35,47,53,58,59,61]
Type 2 diabetes prevention	Partial insulin-independent PPP entry; low glycemic index	Reduced HbA1c and postprandial glucose in animal and human studies	[58,59,60]
Metabolic syndrome/obesity	Lower caloric density than sucrose; anti-inflammatory effects	Animal studies show reduced visceral fat accumulation	[58,60,61]
Oral mucositis	Anti-biofilm; suppresses *P. gingivalis* LPS-induced cytokines; mucosal hydration	Rationale for xylitol wipes/rinses in oncology; phase III trial ongoing	[55,56,66,67,68,69]
Cancer metabolic vulnerability	Glycolytic flux reduction; CHAC1-mediated GSH depletion; ER stress	Reduced proliferation in oral, lung, and melanoma preclinical models	[4,5,6,7,8,9,70,71,72,73]
Cardiovascular risk (indirect)	Reduction in periodontal pathobionts; anti-inflammatory oral environment	Periodontal pathogen reduction; lowered systemic inflammation markers	[22,50,56]

## 9. Evidence That Xylitol Alters Cancer Cell Metabolism

Trachootham et al. showed that partial glucose-to-xylitol substitution reduced proliferation in CAL-27, FaDu, SCC4, SCC9, SCC15, and SCC25 oral squamous carcinoma lines while sparing non-transformed keratinocytes, with reductions in ATP generation and phosphofructokinase (PFK)-linked glycolytic activity [4]. Sahasakul et al. reproduced this in an orthotopic oral tongue xenograft model, in which dietary xylitol substitution prolonged survival and reduced in vivo glycolysis markers [5]. Tomonobu et al. linked xylitol to CHAC1-dependent glutathione depletion, ER stress, oxidative stress, and selective cancer-cell death with chemosensitization in vivo [6]. CHAC1, induced through ATF4-CHOP signaling during ER stress, degrades glutathione and can trigger ferroptosis in a context-dependent manner [71,72]. Park et al. documented xylitol-induced autophagy-associated death in A549 lung cancer cells [7]. Cannon et al. reported early tumor growth delay in B16F10 melanoma syngeneic models with attenuated effects in 4T1 mammary carcinoma [8], and a non-significant trend toward reduced tumor volume with continuous minipump delivery in melanoma, accompanied by metabolomic changes [9] (see Table 3). McCallum and Najlah contextualized these findings within the broader literature on sugar-based metabolic interventions [70]. It is essential to note that the overall body of anticancer evidence for xylitol remains preliminary and largely derived from in vitro cell-culture substrate-substitution studies conducted at millimolar concentrations (typically 1–50 mM) that substantially exceed physiologically achievable systemic levels following oral administration (approximately 0.1–0.5 mM at doses of 20–70 g/day) [47]. Null or attenuated findings include the weak response in the 4T1 syngeneic mammary carcinoma model [8] and should be weighed alongside positive preclinical data. Tumors with high OXPHOS dependence and limited glycolytic flux are unlikely to benefit from this mechanism. Each cited study type (cell culture, animal, human) is indicated parenthetically in Table 3; clinical data on cancer outcomes do not currently exist.

## 10. Cancer Stem Cells and Mitochondrial Adaptation

CSCs shift energy metabolism in response to hypoxia, nutrient stress, therapy, and niche signals [3]. Mitochondrial fission, fusion, mitophagy, and mito-nuclear signaling maintain metabolic flexibility. DRP1-driven fission contributes to proliferation, invasion, and treatment resistance, though context-dependent effects are significant [74]. Zhao et al. linked mitochondrial dynamics and mitophagy to drug resistance [73]. Iwata et al. reviewed strategies targeting mitochondrial structure for synthetic lethality in cancer [44]. Tumor cells can acquire functional mitochondria from stromal and immune cells, restoring OXPHOS capacity after metabolic stress—a process reviewed by Ishino et al. [26]. Mitochondrial DNA release and cGAS-STING activation may either support antitumor immunity or, paradoxically, enhance metastatic fitness depending on recipient-cell context [44]. OPA1-dependent cristae organization is a selective vulnerability in metastatic cells [43]; because XDH within the mitochondrial matrix couples xylitol oxidation to NAD+/NADH cycling, sustained xylitol metabolism could alter ETC activity and cristae-mediated supercomplex stability [16,42].

## 11. Oral Pathogens as Modifiers of Tumor Immunity

*F. nucleatum* promotes tumor stem-cell neogenesis through crypt colonization in colorectal cancer [18], and its outer membrane vesicles activate autophagy and promote oral cancer metastasis [19]. *P. gingivalis* protects oral cancer cells from macrophage killing [21], and its EVs drive invasion in esophageal squamous carcinoma through macrophage-mediated pathways. Lin and colleagues described links between systemic inflammatory disease and macrophage signaling networks [22], and Muñoz-Medel et al. linked *P. gingivalis* to immune evasion in gastric cancer [57]. Shared immune evasion strategies include: TIGIT engagement by Fap2 [20]; M2 macrophage polarization [21,22]; SASP induction in chronically activated immune cells [28,29]; and EMT promotion through secreted bacterial factors [18,19,51]. Xylitol’s anti-adhesive and anti-inflammatory properties may disrupt multiple nodes in this network.

## 12. Integrated Host–Microbe–Mitochondria–CSC Model

Xylitol may create multiple intersecting perturbations: reduction in glycolytic flux and ATP in glucose-dependent tumor cells [4,5]; CHAC1-mediated glutathione depletion and ER/oxidative stress [6,71,72]; reshaping of oral microbial ecology to reduce pathobiont-derived immunosuppressive vesicle production [35,36]; reduction in PAMP-driven immune cell senescence to limit SASP-mediated tumor microenvironment (TME) conditioning [28,29]; and glycemic control that reduces insulin/IGF-1-dependent proliferation signals [62,63]. Compensatory adaptation is expected: tumors may increase reliance on OXPHOS, involving OPA1-dependent cristae reorganization [42,43], or acquire mitochondria from stromal or immune cells [26]. The central question (see Table 4) is whether xylitol’s composite metabolic, antimicrobial, and anti-adhesive properties create synergistic vulnerabilities in defined tumor–microbe–immune contexts. Evidence-level classification of proposed xylitol mechanisms is listed in Table 5. In addition, proposed research relevant to the hypothesis that xylitol may be a context-dependent metabolic modifier within an integrated host–microbe–mitochondria–cancer stem cell network is listed as a potential roadmap for future studies.

## 13. Oral Mucositis and Supportive Cancer Care

Oral mucositis is a clinically significant and dose-limiting toxicity of chemotherapy, radiotherapy, and hematopoietic stem cell transplantation, impairing oral intake, increasing the risk of infection, and precipitating treatment delays or dose reductions [66,67,68]. The MASCC/ISOO guidelines provide evidence-based recommendations built on basic oral care [66,69]. Xylitol is not currently established in these guidelines as a proven mucositis prophylactic, but its anti-cariogenic effects on the biofilm, suppression of *P. gingivalis* LPS-induced cytokines [56], and favorable topical oral-use profile support testing of xylitol-containing wipes, rinses, or lozenges as adjuncts to basic oral care [36]. The NCT07022678 phase III trial of xylitol dental wipes in pediatric AML patients includes severe mucositis and changes in the oral microbiome as exploratory outcomes [55]. Key clinical research gaps that should be addressed to establish evidence-based recommendations include: (a) randomized controlled trials with standardized mucositis grading scales (WHO or OMAS) as primary endpoints; (b) optimal formulation, concentration, and dosing frequency of xylitol-containing rinses or wipes; (c) stratification by cancer type, treatment regimen (chemotherapy versus radiotherapy versus HSCT conditioning), and baseline oral microbiome composition; and (d) assessment of whether xylitol adjuncts reduce secondary infection, treatment interruptions, or analgesic requirement in controlled trials. Establishing such evidence would position xylitol-based interventions within the MASCC/ISOO framework alongside basic oral care protocols.

## 14. Limitations

Key limitations include: (1) most xylitol-cancer studies use cell-culture substrate substitution or local delivery that may not reflect achievable systemic exposures; (2) tumor-type responsiveness is heterogeneous; (3) glucose concentration, osmolarity, and route of administration must be rigorously controlled; (4) microbiome effects cannot be inferred from tumor-cell data alone; (5) anti-adhesive and SASP-limiting effects of xylitol on immune cells require direct experimental validation; and (6) the evolutionary rationale, while reasonable, does not establish population-level cancer-protective benefit. (7) The available literature may be subject to publication bias toward positive preclinical findings. (8) The potential prothrombotic signal associated with elevated circulating xylitol [64] has not been prospectively resolved [65] and represents a possible safety consideration for supplementation at pharmacological doses, particularly in cardiovascular-risk populations. Rigorous next-generation studies should combine stable-isotope tracing, extracellular flux analysis, metabolomics, microbial co-culture, bacterial EV profiling, SASP marker analysis, and live-cell mitochondrial transfer imaging in immunocompetent models.

## 15. Conclusions

Xylitol is a mechanistically very interesting but not yet clinically proven metabolic modifier. Its strongest cancer-relevant evidence involves altered glycolytic flux, ATP production, glutathione/redox biology, ER stress, and autophagy-associated death [4,5,6,7,8,9,70,71,72]. *F. nucleatum*, *P. gingivalis*, and *S. mutans* have established mechanistic links to immune evasion, EMT, stemness, and systemic disease [18,19,20,21,22,51,52]. Oral and gut microbiomes contribute to cancer through metabolite production, immune subversion, and induction of senescent immune cells that drive SASP-mediated tumor progression [23,24,28,29]. Xylitol’s anti-adhesive properties may preserve immune cell longevity by limiting chronic PAMP stimulation. Its metabolic benefits—low glycemic index, insulin-independent hepatic metabolism, and diabetes-prevention potential—intersect with the established metabolic disease–cancer risk axis [60,62,63]. XDH in human mitochondria links xylitol oxidation to NAD+/NADH cycling and cristae-dependent OXPHOS [15,16,42], while the evolutionary adaptation of omnivorous species to polyol-rich diets during MIS 6 underpins human-specific xylitol tolerance [30,31]. Xylitol should be evaluated in defined tumor–microbe–immune contexts. Given its safety profile and composite properties, it may already serve as a beneficial adjunct in oncology oral care for mucositis prevention and microbiome preservation, but must not yet be described as a proven cancer therapy.

## Figures and Tables

**Table 1:** Species-dependent xylitol metabolism and evolutionary context.

Species	XDH Present	Xylitol Metabolism	Dietary Context	Implications
*Homo sapiens*	Yes (liver, mitochondria)	→ D-xylulose → PPP/gluconeogenesis	Omnivore; polyol-rich USOs during MIS 6	Efficient clearance; low toxicity; metabolic benefits [32]
*Rattus norvegicus*	Yes (liver cytosol)	Alditol oxidoreductase pathway; minimal urinary xylitol	Omnivore; frequent metabolic model	High fidelity to humans; early safety studies [32]
*Sus scrofa*	Yes (hepatic)	Hepatic xylulose conversion; insulin-independent	Omnivore; close human GI physiology	Used in dental xylitol research [32]
*Mus musculus*	Minimal	Polyol dehydrogenases	Omnivore; frequent research model	Changes in gut microbiome [46]
*Canis*	Absent or minimal	Accumulates, potent insulin release in dogs	Obligate carnivores; no polyol dietary pressure	Toxic in dogs; hepatotoxicity/hypoglycemia [33]

**Table 3:** Preclinical evidence: xylitol and cancer models.

Model	Principal Observation	Mechanistic Signal	Limitation
Oral SCC lines (CAL-27, FaDu, SCC4, 9, 15, 25) [4]	Xylitol substitution reduced proliferation; keratinocytes spared.	Reduced ATP; lower PFK-linked glycolysis; D-xylulose enhanced suppression.	Physiologic translation unresolved.
Orthotopic oral tongue xenograft [5]	Dietary xylitol prolonged survival and reduced markers of proliferation and glycolysis.	Reduced glycolysis in vivo.	Not a clinical efficacy study.
A549 lung cancer cells [7]	Xylitol inhibited proliferation and induced autophagy-associated death.	Autophagy-linked stress response.	Single cell-line evidence.
CHAC1/GSH models [6,71,72]	Selective cancer-cell death; chemosensitization in vivo.	CHAC1 induction; GSH depletion; ER/oxidative stress.	Concentration and tumor specificity unresolved.
B16F10 melanoma; 4T1 mammary [8]	Early growth delay in B16F10 melanoma; weaker in 4T1 due to interstitial pressure.	Tumor-type-dependent susceptibility.	Heterogeneous immunocompetent model responses.
B16F10 osmotic minipump [9]	Non-significant but reduced mortality and volume trend; significant metabolomic changes.	Redox/energy metabolite shifts.	Pilot; hypothesis-generating only.

**Table 4:** Integrated mechanism and testable implications.

Axis	Evidence-Supported Observation	Testable Implication
Warburg/lactate axis	Tumors use aerobic glycolysis with intact mitochondria; lactate and lactylation modulate immunity [1,2,27,39,40,41].	Quantify glycolytic flux, lactate, and lactylation under xylitol exposure.
Xylitol metabolic perturbation	Reduced ATP, glycolysis markers, GSH; ER/oxidative stress; altered metabolomics [4,5,6,7,8,9,71,72].	Dose–response designs with matched glucose/osmolarity controls.
CSC plasticity	CSC states exploit mitochondrial remodeling and metabolic switching to tolerate stress [3,73,74].	Test in tumorspheres, CSC-enriched populations, and therapy-resistant models.
Oral pathobionts	*F. nucleatum*, *P. gingivalis*, and *S. mutans* drive immune evasion, EMT, and vesicle-mediated metastasis [18,19,20,21,22,51,52].	Microbial co-culture, EV exposure, microbiome endpoints in oral cancer models.
Immune cell senescence/SASP	Chronic pathobiont stimulation drives immune cell senescence, and the SASP promotes tumor progression [28,29].	SASP profiling in immune cells ± xylitol; NK/T cell longevity assays.
Mitochondrial transfer	Tumor cells acquire mitochondria from immune/stromal cells, restoring OXPHOS [26].	Quantify mitochondrial transfer; test dependence on transfer-mediated adaptation.
Metabolic disease/glycemic axis	Hyperglycemia, hyperinsulinemia, IGF-1 promote cancer via PI3K/mTOR [62,63].	Xylitol as a glucose substitute in hyperglycemic cancer models.
Anti-adhesion axis	Xylitol blocks pathogen adhesion to host receptors, reducing PAMP-driven SASP [37,53,54].	Pathobiont adhesion to immune cells ± xylitol; SASP marker quantification.

**Table 5:** Evidence-level classification for proposed xylitol mechanisms.

Proposed Mechanism	Best Supporting Evidence	Evidence Level	Key Caveat/Limitation	Clinical Gap/Next Step
Xylitol reduces glycolytic flux and ATP in oral SCC lines	Multiple oral SCC cell lines (Trachootham et al. [4]); orthotopic xenograft (Sahasakul et al. [5])	**C**—In vitro/preclinical in vivo	Concentrations exceed physiologically achievable systemic levels; no clinical cancer outcome data	Dose–response studies with matched osmolarity controls; stable-isotope tracing
CHAC1-mediated GSH depletion and ER stress in cancer cells	In vitro + in vivo chemosensitization (Tomonobu et al. [6,71,72])	**B/C**—Preclinical	Tumor-type and concentration specificity unresolved; ferroptosis context-dependent	Cancer-type panel with XDH expression stratification; GSH/ROS flux assays
Xylitol inhibits *S. mutans* virulence and adhesion	Multiple clinical trials; caries reductions of 30–60% (Mäkinen et al. [47]); maternal transmission (Söderling [53])	**A**—Clinical RCT	Anti-caries evidence is strong; direct anti-tumor outcome from *S. mutans* suppression not yet demonstrated	Microbiome sequencing endpoints in oncology oral care trials
Xylitol inhibits *P. gingivalis* and *F. nucleatum* adhesion/cytokine induction	In vitro (Han et al. [56]; Cannon & Stevenson [35]); LPS-induced cytokine suppression in macrophages	**C**—In vitro	Anti-tumor outcomes from pathobiont suppression in humans not demonstrated	EV exposure/microbial co-culture in oral cancer models; SASP marker quantification
Xylitol limits immune cell senescence and SASP via anti-adhesion to immune receptors	Mechanistic inference from anti-adhesion data (Kontiokari et al. [54]) and SASP biology (Campisi et al. [28,29])	**E**—Theoretical hypothesis	No direct experimental evidence; proposed mechanism only	SASP profiling in macrophages/neutrophils ± xylitol; NK/T cell longevity and cytotoxicity assays
Xylitol metabolism (XDH) modulates mitochondrial cristae/OPA1/OXPHOS supercomplexes	Indirect: XDH alters NADH/NAD+ [16]; cristae shape OXPHOS supercomplexes (Cogliati et al. [42]); OPA1 vulnerability in metastatic breast cancer (Diokmetzidou et al. [43])	**D/E**—Indirect/theoretical	No direct evidence linking xylitol to cristae ultrastructure in cancer cells	Seahorse OCR/ECAR; TEM for cristae ultrastructure; BN-PAGE for ETC supercomplex profiling; U-13C5-xylitol flux tracing
Xylitol reduces metabolic disease risk, interrupting insulin/IGF-1-driven cancer promotion	Human/animal data on HbA1c and postprandial glucose reduction (Islam [60]); metabolic disease–cancer link established (Cowey & Hardy [62]; Gallagher & LeRoith [63])	**D**—Indirect inference	No prospective trial linking xylitol use to reduced cancer incidence	Xylitol as glucose substitute in hyperglycemic cancer models; cancer risk endpoints in diabetes-prevention trials
Xylitol as mucositis adjunct in oncology oral care	Phase III trial ongoing (NCT07022678, pediatric AML); anti-biofilm and cytokine suppression data [55,56,66,69]	**B/D**—Preclinical + indirect	Not in current MASCC/ISOO guidelines; RCT evidence lacking	RCTs with WHO/OMAS mucositis grading; stratify by cancer type, treatment regimen, baseline microbiome
Xylitol supplementation—cardiovascular safety	Witkowski et al. [64]: elevated circulating xylitol associated with platelet aggregation and CV events; exogenous xylitol enhanced platelet reactivity ex vivo and in mice	**D**—Observational/mechanistic (no RCT)	Endogenous xylitol signal; pharmacological doses used; confounding by metabolic disease not excluded	Prospective RCT powered for CV safety endpoints at dietary doses (2–40 g/day); distinguish endogenous vs. exogenous xylitol contribution

Evidence levels: A = Clinical RCT or prospective cohort • B = Preclinical in vivo • C = In vitro • D = Indirect mechanistic inference • E = Theoretical hypothesis. Abbreviations: BN-PAGE = blue native PAGE; EV = extracellular vesicle; GSH = glutathione; MASCC/ISOO = Multinational Association of Supportive Care in Cancer/International Society of Oral Oncology; OCR/ECAR = oxygen consumption rate/extracellular acidification rate; OPA1 = optic atrophy protein 1; OXPHOS = oxidative phosphorylation; PAMP = pathogen-associated molecular pattern; RCT = randomized controlled trial; SASP = senescence-associated secretory phenotype; SCC = squamous cell carcinoma; TEM = transmission electron microscopy; XDH = xylitol dehydrogenase.

## Data Availability

No new data were created or analyzed in this study. Data sharing is not applicable.

## Abbreviations

AML	acute myeloid leukemia
ATP	adenosine triphosphate
CHAC1	ChaC glutathione-specific gamma-glutamylcyclotransferase 1
cGAS-STING	cyclic GMP-AMP synthase–stimulator of interferon genes
CSC	cancer stem cell
DRP1	dynamin-related protein 1
ER	endoplasmic reticulum
ETC	electron transport chain
EV	extracellular vesicle
EMT	epithelial–mesenchymal transition
GSH	glutathione
GLUT2	glucose transporter 2
GLUT4	glucose transporter 4 (insulin-dependent)
IGF-1	insulin-like growth factor 1
MIS 6	Marine Isotope Stage 6
MICOS	mitochondrial contact site and cristae organizing system
NK	natural killer
OPA1	optic atrophy protein 1
OXPHOS	oxidative phosphorylation
PAMP	pathogen-associated molecular pattern
PFK	phosphofructokinase
PPP	pentose phosphate pathway
ROS	reactive oxygen species
SASP	senescence-associated secretory phenotype
TME	tumor microenvironment
USO	underground storage organ
XDH	xylitol dehydrogenase
